# Medium-chain carboxylates production from plant waste: kinetic study and effect of an enriched microbiome

**DOI:** 10.1186/s13068-024-02528-y

**Published:** 2024-06-12

**Authors:** Jerome Undiandeye, Daniela Gallegos, Maria L. Bonatelli, Sabine Kleinsteuber, Mohammad Sufian Bin-Hudari, Nafi’u Abdulkadir, Walter Stinner, Heike Sträuber

**Affiliations:** 1 Department of Biochemical Conversion, DBFZ Deutsches Biomasseforschungszentrum Gemeinnützige GmbH, 04347 Leipzig, Germany; 2 Department of Chemical Engineering, University of Port Harcourt, PMB 5323, Port Harcourt, Nigeria; 3 Department of Microbial Biotechnology, Helmholtz Centre for Environmental Research – UFZ, 04318 Leipzig, Germany; 4 Department of Isotope Biogeochemistry, Helmholtz Centre for Environmental Research – UFZ, 04318 Leipzig, Germany; 5 Research Unit for Comparative Microbiome Analysis, Helmholtz Munich, German Research Center for Environmental Health, 85764 Neuherberg, Germany; 6 Department of Microbiology, Sokoto State University, Sokoto, 852101 Nigeria

**Keywords:** Anaerobic fermentation, Caproate, Electron donors, Lactate, Microbial chain elongation, Waste biomass

## Abstract

**Background:**

The need for addition of external electron donors such as ethanol or lactate impairs the economic viability of chain elongation (CE) processes for the production of medium-chain carboxylates (MCC). However, using feedstocks with inherent electron donors such as silages of waste biomass can improve the economics. Moreover, the use of an appropriate inoculum is critical to the overall efficiency of the CE process, as the production of a desired MCC can significantly be influenced by the presence or absence of specific microorganisms and their metabolic interactions. Beyond, it is necessary to generate data that can be used for reactor design, simulation and optimization of a given CE process. Such data can be obtained using appropriate mathematical models to predict the dynamics of the CE process.

**Results:**

In batch experiments using silages of sugar beet leaves, cassava leaves, and *Elodea*/wheat straw as substrates, caproate was the only MCC produced with maximum yields of 1.97, 3.48, and 0.88 g/kgVS, respectively. The MCC concentrations were accurately predicted with the modified Gompertz model. In a semi-continuous fermentation with ensiled sugar beet leaves as substrate and digestate from a biogas reactor as the sole inoculum, a prolonged lag phase of 7 days was observed for the production of MCC (C6–C8). The lag phase was significantly shortened by at least 4 days when an enriched inoculum was added to the system. With the enriched inoculum, an MCC yield of 93.67 g/kgVS and a productivity of 2.05 gMCC/L/d were achieved. Without the enriched inoculum, MCC yield and productivity were 43.30 g/kgVS and 0.95 gMCC/L/d, respectively. The higher MCC production was accompanied by higher relative abundances of Lachnospiraceae and Eubacteriaceae*.*

**Conclusions:**

Ensiled waste biomass is a suitable substrate for MCC production using CE. For an enhanced production of MCC from ensiled sugar beet leaves, the use of an enriched inoculum is recommended for a fast process start and high production performance.

**Supplementary Information:**

The online version contains supplementary material available at 10.1186/s13068-024-02528-y.

## Background

Even-numbered medium-chain carboxylates (MCCs) containing 6–12 carbon atoms are mainly produced as by-products of palm oil refining [1]. The demand for MCCs has been steadily increasing, and the global market size for these products is forecasted to reach USD 2.7 billion by 2027 [2]. MCCs have been reported to have a higher economic value than biogas, bioethanol, or biodiesel [3]. They are used in industries as feed additives [4], inhibitors of microbial corrosion [5] or for the production of lubricants [6]. A process commonly referred to as microbial chain elongation (CE), which is part of anaerobic fermentation, uses electron donors, usually ethanol or lactate, and electron acceptors such as acetate [7] for the production of MCCs, such as caproate (C6) and caprylate (C8). CE processes are catalyzed by anaerobic bacterial consortia and are basically redox reactions that follow the reverse β-oxidation pathway [8].

For a sustainable production of MCC, it is necessary to use substrates whose generation does not compete with food production. Such substrates include agricultural residues, such as sugar beet leaves or cassava leaves, as well as aquatic plants, such as *Elodea*. Sugar beet leaves are a by-product of sugar beet harvesting and account for 25–30% of the fresh sugar beet crop. At least 120 million tons of sugar beet leaves are produced annually in Europe [9], of which an estimated 18 million tons are produced in Germany. On the other hand, Nigeria produced 59.19 million tons of cassava in 2019 [10], accounting for 19.47% of the global cassava production in that year. One of the wastes usually generated during the harvesting of cassava root is cassava leaves, which can contain cyanide in high concentration [11], making them unsuitable for use as animal feed. *Elodea*, an invasive water plant, has been described as a global ecological problem that can often only be addressed by removal from water bodies [12]. The availability of these three kinds of plant waste makes them potential substrates for the production of MCCs by anaerobic fermentation, thereby reducing their potential environmental impact. Although these biomasses are easily degradable, they can be preserved by ensiling [13]. During ensiling, lactate and ethanol are formed that could be used as electron donors in subsequent MCC production [8], thus avoiding the need to add them to the process. Moreover, ensiling ensures year-round substrate availability and consistent quality, and serves as a pre-treatment technique [14].

For the start of a new MCC production process, the use of an inoculum can be helpful. An inoculum from a biogas reactor may be adequate for starting CE processes, and working under non-sterile conditions could reduce overall costs. However, the use of such an inoculum could be associated with a long lag-phase of CE and limit the formation of MCCs [15] if the abundance of key community members, such as *Caproiciproducens*, which have been reported to correlate positively with caproate formation [16], is limited. One way to improve the CE efficiency could be the addition of broth from a previous or an already running CE process as co-inoculum or for bioaugmentation.

The yield and production rates of even- and odd-numbered carboxylates in an anaerobic fermentation process depend on the composition and dynamics of the microbial community, which in turn is influenced by the concentration and nature of the substrate, as well as process parameters, such as temperature and pH [7, 8]. Insight into the metabolic functions of community members with their various metabolic pathways enables the identification of process parameters that favor the production of specific MCCs. Using mathematical models, the understanding of the anaerobic fermentation process can be expanded. The models can provide kinetic parameters required for the design, operation, and optimization of processes in bioreactors to enhance their efficiency [17]. In addition, they can deliver information on the rate of a microbial process as a function of the operating parameters, enabling the determination of optimal conditions for the production of a specific MCC. While kinetic models for CE have been developed for pure cultures and model substrates, such as glucose [18], only a few studies on kinetic modeling of anaerobic fermentation of complex substrates by mixed microbial cultures have been reported.

The objectives of this study were to (*i*) investigate the suitability of selected silages for MCC production and determine kinetic parameters, (*ii*) study the effect of a mixture of an enriched microbiome and inoculum from a biogas reactor on the lag-phase of MCC production and the MCC yield, and (*iii*) identify the microbial community involved in the production of MCCs from ensiled sugar beet leaves using different inocula.

## Materials and methods

### Substrates and inocula

Four silages from different plant biomasses and one chemical standard were used as substrates. Sugar beet leaves were obtained from a local farm in Saxony, Germany, and ensiled as previously described [19]. Cassava leaves obtained from Ogoja, Southern Nigeria, were washed, grinded using a kitchen blender, packed into airtight bags and vacuum sealed. Silage consisting exclusively of *Elodea* biomass could not be prepared due to its high water content. Instead, a mixture of *Elodea* and wheat straw, ensiled as described by Gallegos et al. [20], was obtained from Deutsches Biomasseforschungszentrum (DBFZ). Maize silage was also obtained from the DBFZ. Xylan (X) and lactate (L), which were used as chemical standard substrates, were obtained from Sigma Aldrich. The physico-chemical properties of all substrates, except xylan and lactate, are listed in Table 1.

**Table 1:** Mean values (± standard deviation) of the physico-chemical properties of substrates

Parameter	Unit	Substrates			
		MS	SBL	Cass	Elo
Total solids	%FM	38.90 ± 3.68	16.14 ± 0.16	24.29 ± 0.07	24.17 ± 0.10
Volatile solids	%TS	96.93 ± 0.01	73.70 ± 0.20	91.06 ± 0.04	91.76 ± 0.12
pH	–	3.86 ± 0.01	4.71 ± 0.22	4.76 ± 0.04	3.94 ± 0.01
Acetate	g/kgFM	77.56 ± 0.00	42.93 ± 0.01	26.73 ± 0.00	56.14 ± 0.00
Propionate	g/kgFM	8.73 ± 0.00	0.22 ± 0.00	ND	2.98 ± 0.00
Butyrate	g/kgFM	5.64 ± 0.00	0.09 ± 0.00	0.12 ± 0.00	1.45 ± 0.00
Caproate	g/kgFM	0.16 ± 0.00	0.02 ± 0.00	0.02 ± 0.00	ND
Lactate	g/kgFM	286.26 ± 9.68	103.29 ± 6.01	111.60 ± 9.73	81.11 ± 7.24
Ethanol	g/kgFM	10.07 ± 0.00	15.32 ± 0.04	23.83 ± 0.01	0.65 ± 0.00

MS, maize silage; SBL, sugar beet leaf silage; Cass, cassava leaf silage; Elo, *Elodea*/wheat straw silage; FM, fresh mass; TS, total solids; ND, not detected

Three different inocula were used for anaerobic fermentation. Inoculum 1 and 2 were obtained from two different anaerobic digesters at the DBFZ. Inoculum 1 (total solids, TS, 6.87%; volatile solids, VS, 72.18%TS; pH 7.57) was used for batch fermentation and originated from an anaerobic continuous stirred tank reactor (organic loading rate, OLR, 0.5 gVS/L/d; hydraulic retention time, HRT, 100 d; 38 °C) that was fed with a substrate mixture consisting of maize silage, cattle manure, and sun flower oil. Inoculum 2 (TS, 3.44%; VS, 70.23%TS; pH 7.0) was used for the semi-continuous fermentation of sugar beet leaf silage and was obtained from a full-scale biogas plant operated with a mixture of maize silage and cattle manure (HRT, 37 d; OLR, 4 gVS/L/d; 38 °C). Before being used, Inoculum 2 was sieved to remove large substrate particles using a mesh (2 mm). An enriched microbiome from the anaerobic fermentation of maize silage described in a previous study [21] was used as source for Inoculum 3. It was first centrifuged at 10,000 × *g* for 10 min. The supernatant was then removed and the obtained pellet was washed with deionized water. The pellet obtained from 3 L of the enriched microbiome was resuspended in 1.2 L deionized water and used as Inoculum 3. All inocula were pre-treated for 1 h at 90 °C with the aim to inactivate methanogens.

### Batch fermentation in serum bottles

Batch fermentations were set up in duplicates with silages of sugar beet leaves, cassava leaves, or *Elodea*/wheat straw mixture in identical 200 mL serum bottles with working volumes of 125 mL. The empty bottles were transferred into an anaerobic chamber, where the substrates and Inoculum 1 were added. Each of the bottles contained 2.72 gVS of the respective substrate, 100 mL (4.91 gVS) of inoculum and, if necessary, anoxic deionized water to achieve the stated working volume. For comparison, bottles containing a standard plant substrate (maize silage) and bottles with a chemical standard substrate consisting of a mixture of xylan and lactate (xylan:lactate = 3:1 *w/w*) were set up. Substrate-free control bottles containing only water and inoculum were also prepared. The pH in each bottle was adjusted to 7.57 ± 0.02 (the pH of the inoculum) using 10 M sodium hydroxide solution. The bottles were closed with butyl rubber stoppers and aluminium caps, removed from the anaerobic chamber, and incubated at 38 °C and 100 rpm for 28 days. To reduce the pressure in the bottles caused by gas production, each system was depressurized to 0.009 (± 0.002) bar(g) after each sampling.

### Semi-continuous fermentation in reactors

Two 15-L stirred tank reactors (STR) named R7 and R8 (Bräutigam Kunststoffsysteme GmbH), each with a working volume of 12 L, equipped with an overhead stirrer (RZR2102, Heidolph Instruments) and operated in semi-continuous mode, were used for the anaerobic fermentation of sugar beet leaf silage in two trials. In the first experiment (E1), only Inoculum 2 was used, while a mixture of Inoculum 2 and Inoculum 3 was used in the second experiment (E2). Based on a previous study [22], the pH of the reactors was set at 5.50 ± 0.02 and then controlled automatically in both experiments using a feedback controller consisting of a pH sensor (pH 3110, WTW, Xylem Analytics Germany Sales GmbH & Co. KG), a control unit (Inpro 325X, Mettler Toledo) and a pump (Pumpdrive 5201, Heidolph Instruments) connected to each reactor and dispensing 10 M NaOH solution. The temperature was maintained at 38 °C (MA-4 Umwälzthermostat, Julabo). To compensate for pressure fluctuations during feeding, two 5-L gas bags were connected to the headspace of each reactor. The HRT was set to 4 days and the OLR was set to 21.9 gVS/L/d for both experiments.

For E1, the initial reactor content consisted of 6 L of Inoculum 2 and 3 L of substrate mixture. For E2, 3 L of each of Inoculum 2 and 3, and 3 L of substrate mixture were added to each reactor. For both E1 and E2, the headspaces of the reactors were flushed with N_2_ to provide anaerobic conditions. Until the desired working volume of 12 L was reached, the reactors were operated in fed-batch mode without withdrawal of fermentation broth. Thereafter, 3 L of substrate mixture containing an appropriate amount of deionized water and substrate as well as 3 mL of trace element solution with a previously described composition [8] and 18 g of ammonium bicarbonate as additional nitrogen source (NH_4_HCO_3_; Carl Roth) were fed to each reactor daily. Before feeding, 3 L of fermentation broth was harvested from the reactors to keep the working volume constant.

### Process analytics

TS and VS contents of substrates and fermentation broths were determined according to standard methods [23], and the values were corrected for the loss of organic acids and alcohols according to Weißbach and Strubelt [24] to avoid over-estimating the product yields and conversion degrees. The pH of samples was measured using a pH meter (model 3310; WTW, Xylem Analytics Germany Sales GmbH & Co. KG) equipped with a Sentix 41 pH electrode (WTW). Concentrations of carboxylates and ethanol were measured using a 7890A gas chromatograph with a flame ionization detector (FID; Agilent Technologies) as described by Apelt [25]. The head space of all reactors was sampled and the gas composition was measured with a gas chromatograph (Perkin Elmer) equipped with an auto-sampler following the method of Sträuber et al. [22].

To determine the gas production in the serum bottles, a digital manometer (LEO 5; Keller) connected to a filter (pore size 0.20 µm; Labsolute) was used to measure the relative pressure in the bottles. The amount of gas components produced in the bottles was calculated using the modified ideal gas equation shown in the following equation:

1 $${Gas}_{\left({x}_{i}\right)}\left(mmol\right)=\frac{{P}_{abs}\times \frac{{Gas}_{\left(xi\right)}\left(\%\right)}{100}\times {V}_{h}}{R\times T}\times 1000$$

where *xi* is the gas component in the gas mixture, *P*_*abs*_ is the absolute pressure in the bottle (in mbar), *V*_*h*_ is the volume of the head space inside the bottle (75 mL), *R* is the universal gas constant (83.140 mbar cm^3^/mol/K), and *T* is the gas temperature (K).

The gas production in the STRs was measured using Milligas counters MGC-IV3.1 (Ritter Apparatebau) connected to each reactor. The measured values were normalized to standard conditions as described previously [22]. Ammonia–nitrogen (NH_3_–N) content in harvested fermentation broths from E1 and E2 was measured using ammonia test kits, Nessler reagent and a spectrophotometer (Hach DR 2000; HACH LANGE GmbH, Germany) according to the method described by Strach [26].

The yield of a fermentation product, P (*Y*_*P/S*_; given in g_Product_ per kgVS_Substrate_) in the batch systems and STRs was calculated using Eqs. 2 and 3, respectively. The selectivity of a carboxylate, P in both batch and STRs, was calculated using Eq. 4:

2 $${Y}_{P/S}=\frac{{\rho }_{P}\times V}{{m}_{S}}$$

3 $${Y}_{P/S}=\frac{{\rho }_{P}\times \vartheta }{{m}_{s}}$$

4 $${S}_{P}=\left(\frac{{\rho }_{P}}{{\rho }_{T}}\right)\times 100\%$$

where *ρ*_*P*_ is the mass concentration of the product in the fermentation broth (g/L), *V* is the working volume in the bottles (= 0.125 L), *m*_*S*_ is the mass of added substrate (kgVS), $\vartheta$ is the volume of fermentation broth daily withdrawn from the STRs (= 3 L), S_P_ is the selectivity, and ${\rho }_{T}$ is the total mass concentration of carboxylates in the fermentation broth (g/L).

### Microbial community analysis

Cell pellets of the fermentation broths from E1 and E2 were collected at different timepoints and washed with 12 mM phosphate-buffered saline pH of 7.4 [27]. The cells were then stored at -20 °C until further processing. DNA was extracted from frozen pellets using the NucleoSpin Soil Kit (Macherey–Nagel). DNA quality was checked using a NanoDrop® ND-1000 UV–Vis spectral photometer (Fisher Scientific) and agarose gel electrophoresis; DNA concentration was determined with the Qubit dsDNA BR Assay-Kit (Invitrogen). Microbial community analysis was performed by amplicon sequencing of 16S rRNA genes. Library preparation and sequencing on the Illumina MiSeq platform were done as described by Logroño et al. [28].

Data were processed with DADA2 v.1.28.0 [29] to infer the amplicon sequence variants (ASVs). The R packages phyloseq v.1.44.0 and vegan v. 2.6.4 were used to perform the compositional and statistical analyses [30, 31] on R software v.4.3.0 [32]. The SILVA database (version 138.1; [33]) was used for taxonomic assignment of ASVs. Raw sequence data were deposited at the NCBI database under the study accession PRJNA926909.

### Modeling

Four kinetic models were used to describe the production of propionate, butyrate and caproate during the batch fermentation processes. These models were the first-order model given in Eq. 5, the modified Gompertz model given in Eq. 6, the Logistic model given in Eq. 7, and the Fitzhugh model given in Eq. 8, using the Solver add-in program in Microsoft Excel with sum of squared errors as an objective function:

5 $${\rho }_{t}={\rho }_{max}(1-\text{exp}\left(-kt\right))$$

6 $${\rho }_{t}={\rho }_{max}\times \text{exp}(-\text{exp}\left(\frac{{R}_{max}\times e}{{\rho }_{max}}\left(\lambda -t\right)+1\right))$$

7 $${\rho }_{t}=\frac{{\rho }_{max}}{1+exp\left[\frac{4\times {R}_{max}(\lambda -t)}{{\rho }_{max}}+2\right]}$$

8 $${\rho }_{t}={\rho }_{max}\left[{1-\text{exp}(-kt)}^{n}\right]$$

where ${\rho }_{t}$ is the concentration of a given organic acid at timepoint t (g/L), ${\rho }_{max}$ is the maximum possible concentration of a given organic acid (g/L), *k* is the first-order carboxylate production rate constant (/d), *R*_*max*_ is the maximum carboxylate production rate (g/L/d), *λ* is the process lag-phase (d), *e* is the Euler’s constant, and n is the shape constant.

The model that fitted the experimental data best was identified using the coefficient of determination (R^2^), the Akaike Information Criterion (AIC) given in Eq. 9 and the root-mean-square-error given in Eq. 10:

9 $$AIC=N\times ln\left(\frac{ss}{N}\right)+2v$$

10 $$RMSE=\sqrt{\frac{ss}{N}}$$

where *N* is the number of experimental data; *ss* is the square sum of residuals; and *v* is the number of parameters in the model.

All comparisons among substrates were performed using a one-way ANOVA followed by Tukey’s post hock test at 0.05 significance level using SAS v 10.0 software (SAS institute). Pearson correlation analysis was used to evaluate the interdependence of fermentation products using the same statistical software.

## Results and discussion

### Anaerobic fermentation in the batch systems

Batch fermentations for the production of short-chain carboxylates and MCC from silages of maize, sugar beet leaves, cassava leaves and *Elodea*/wheat straw, as well as from the model substrate xylan + lactate were carried out using Inoculum 1. The production yields and selectivities of acetate, propionate, butyrate and caproate were significantly different (p < 0.05) after 21 days (Table 2), probably due to the different physico-chemical properties of the individual substrates, in particular the contents of the electron donors lactate and ethanol (Table 1). Also, the proportion of lignocellulose and thus the accessibility and share of fermentable components such as hemicellulose varied between the different substrates (hemicellulose contents: 4.66%TS [34], 12.6%TS [35], 10.78%TS [13], and 28.3%TS [34] in *Elodea*, cassava leaves, sugar beet leaves, and wheat straw, respectively).

**Table 2:** Maximum yields and selectivities (± standard deviation) of carboxylates after 21 days of batch fermentation

		Substrates				
		X + L	MS	SBL	Cass	Elo
Yield (g/kgVS)	C2	216.23 ± 0.09	258.69 ± 7.54	303.56 ± 3.21	408.65 ± 1.49	176.207 ± 1.94
	C3	46.55 ± 0.07	14.46 ± 4.82	33.99 ± 1.38	30.97 ± 1.21	10.11 ± 0.25
	C4	265.54 ± 2.45	78.69 ± 1.49	24.89 ± 1.42	66.19 ± 1.17	10.88 ± 1.63
	C5	4.85 ± 0.83	10.56 ± 1.93	1.07 ± 0.03	8.80 ± 0.12	3.27 ± 0.07
	C6	12.21 ± 1.56	21.03 ± 13.11	1.97 ± 0.01	3.48 ± 0.05	0.88 ± 0.01
Selectivity (%)	C2	51.69 ± 1.77	66.09 ± 7.74	85.90 ± 0.79	81.73 ± 3.69	90.79 ± 3.11
	C3	7.46 ± 0.19	4.29 ± 0.86	8.12 ± 1.52	5.65 ± 0.23	4.44 ± 0.27
	C4	34.47 ± 0.94	13.48 ± 0.41	5.28 ± 0.36	10.65 ± 0.18	3.89 ± 0.03
	C5	0.68 ± 0.09	1.76 ± 0.23	0.24 ± 0.06	1.27 ± 0.07	0.66 ± 0.01
	C6	1.60 ± 0.14	3.39 ± 1.96	0.42 ± 0.01	0.68 ± 0.02	0.18 ± 0.01

C2, acetate; C3, propionate; C4, butyrate; C5, valerate; C6, caproate; X + L, xylate + lactate; MS, maize silage; SBL, sugar beet leaf silage; Cass, cassava leaf silage; Elo, *Elodea*/wheat straw silage

Acetate was the predominant fermentation product (83.00% from sugar beet leaves, 78.85% from cassava leaves, and 87.40% from *Elodea*/wheat straw), which is consistent with other studies [15, 36], where digestates from biogas reactors were also used as inoculum, probably due to the high abundance of acetate-forming bacteria in the inoculum.

**Fig. 1:**
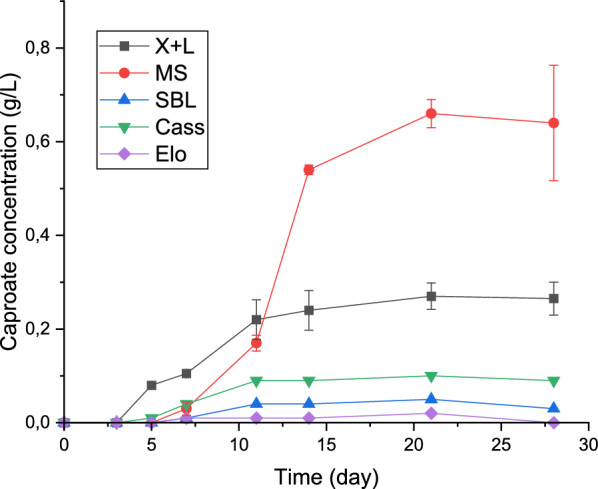
Dynamics of caproate production from the batch fermentation of the substrates (X + L, xylate + lactate mixture; MS, maize silage; SBL, sugar beet leaf silage; Cass, cassava leaf silage; Elo, *Elodea*/wheat straw silage). Error bars show the standard deviation of biological replicates

MCCs such as caproate were not produced in any of the bottles until day 7 (Fig. 1). This was in accordance with Liu et al. [37], who reported that the time for caproate to be detected in batch fermentation of lactate can vary between 1 and 7 days, depending on the bacterial composition of the inoculum. The concentration of caproate increased gradually in all bottles, reaching a maximum of 0.64, 0.02, 0.05, 0.08, and 0.27 g/L in the systems with maize silage, *Elodea*/wheat straw silage, sugar beet leaf silage, cassava leaf silage and xylan + lactate, respectively, on day 21. Although enanthate (C7; 0.10 g/L) and caprylate (C8; 0.30 g/L) were produced in the bottles containing maize silage, the only MCC detected in all other bottles was caproate. Lactate and ethanol were completely consumed in all bottles, probably because they were used for the formation of carboxylates. According to Tang et al. [16], a high concentration of an electron donor like lactate can lead to increased butyrate and caproate production during anaerobic fermentation. Consequently, more caproate was produced from substrates with higher lactate concentration in the present study (Fig. 1). This emphasizes the need to ensure proper ensiling of substrates that are to be used for MCC production. In the bottles with the chemical standard xylan + lactate, the caproate production remained low, despite high butyrate production (Table 2). It is likely that Inoculum 1 had low ability for microbial CE of butyrate or that the conditions were not suitable for this step.

A decrease in pH was observed in all bottles due to the formation of carboxylates (Figure S1). The pH drop was more pronounced during the first 3 days of fermentation, after which the pH remained relatively stable. This pattern was similarly observed by Fang et al. [38] during the anaerobic fermentation of mushroom residue. The pH decreased more in the order of systems containing silages of cassava leaves, sugar beet leaves and *Elodea*/wheat straw, which corresponded to the systems with higher carboxylate yields (Table 2), which was also observed by Eryildiz et al. [39].

Methane was not detected in any of the bottles in the first week, until traces of the gas were found in the bottles containing *Elodea*/wheat straw silage on day 7. On day 11, all other bottles except those with xylan + lactate contained methane. The gradual increase in methane production in the bottles indicates that the methanogens were only partially inactivated by the heat treatment of the inoculum or that methanogens were added with the non-sterile silages. The methane yields of the systems containing silages of cassava leaves, sugar beet leaves, *Elodea*/wheat straw and maize were 171, 196, 609 and 6 mL/gVS, respectively, on day 21. Of the systems with silages, those that produced more methane produced less caproate, indicating the competitive relationship between methanogenesis and CE.

In all bottles throughout the experimental period, the hydrogen partial pressure was below the value of 304 kPa required for a sustained caproate production [40], probably because the bottles were degassed after each analysis. The low hydrogen partial pressure may also have enhanced the excessive oxidation of ethanol to acetate [41].

### Kinetics of carboxylate production in batch systems

**Fig. 2:**
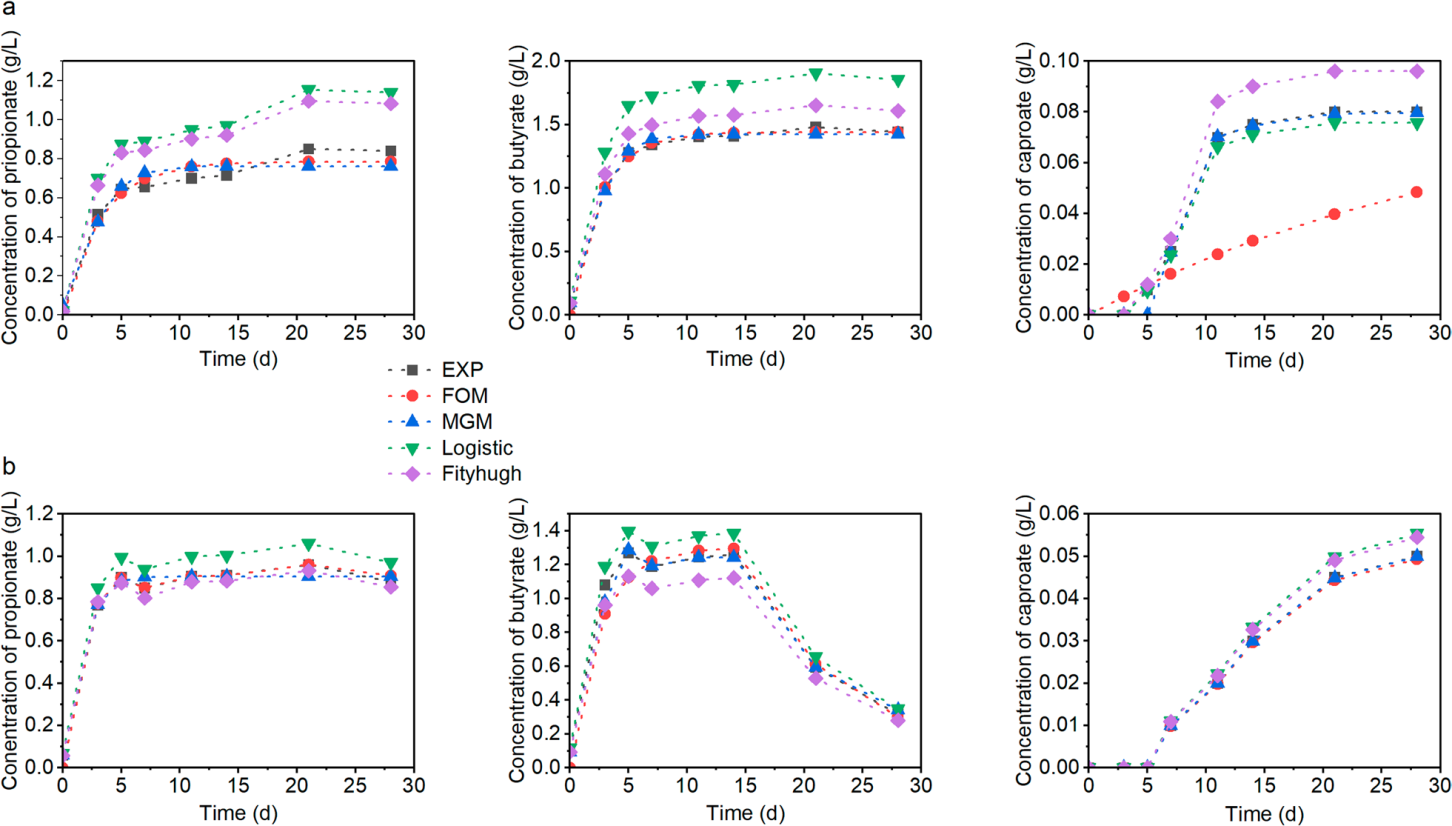
Fitting curves from kinetic modeling of propionate, butyrate and caproate formation from the fermentation of silages of cassava leaves (**a**) and sugar beet leaves (**b**) using the first order model (FOM), the modified Gompertz model (MGM), the Logistic model and the Fitzhugh model

Since the formation of propionate from lactate could be a competing pathway to the production of caproate by CE [39, 42], its kinetic parameters were determined in addition to those of butyrate and caproate production. The experimental values were fitted to four kinetic models, and various kinetic parameters were estimated using the Solver add-in program in Microsoft Excel. The estimated parameters and the fit of the measured data to the models are shown in Table 3 and Fig. 2, respectively. Most of the selected models adequately predicted propionate, butyrate and caproate production in all systems except in the bottles containing *Elodea*/wheat straw where only propionate production could be predicted by the models. The adequate prediction of the carboxylates production by the models is seen from the high values of R^2^, which were between 0.847 and 1.000. However, amongst the models used to describe the kinetics of propionate production, the first-order model gave the best prediction as indicated by the lowest values of AIC and RMSE. The model allowed for the estimation of a propionate production rate constant (k) of 0.115, 0.123 and 0.089/d in the system containing the silages of cassava leaves, sugar beet leaves and *Elodea*/wheat straw, respectively. These values were lower but not significantly different (p > 0.05) from 0.117, 0.126 and 0.103/d obtained from the Fitzhugh model for the silages of cassava leaves, sugar beet leaves and *Elodea*/wheat straw, respectively. Such discrepancy in kinetic parameters from different models could be attributed to the assumptions made in the derivation of the respective models. Morais et al. [43] and Coelho et al. [44] have also reported such differences in their studies. With lower AIC and RMSE values than the other models, the modified Gompertz model predicted butyrate production from the silages of cassava leaves and sugar beet leaves better than the other models. However, butyrate production from the system containing *Elodea*/wheat straw was too low to be predicted by any of the models. A lag phase of 0 days was predicted by both the modified Gompertz and the Logistic model for the production of propionate and butyrate from all substrates, with the shape factor of the Fitzhugh model being less than 1.0 for propionate and butyrate formation. A shape factor of less than 1 is an indication of the absence of a lag phase.

**Table 3:** Kinetic and statistical parameters of carboxylate production from different silages determined by modeling

Model	Parameter	Cassava leaf silage			Sugar beet leaf silage		
		C3	C4	C6	C3	C4	C6
	k (/d)	0.115	0.129	–	0.123	0.119	–
First order	R^2^	0.964	0.997	0.847	0.999	0.969	1.000
	AIC	− 46.529	− 50.542	− 54.341	− 57.995	− 36.484	− 123.724
	RMSE	0.048	0.038	0.030	0.024	0.090	0.000
	R_max_ (g/L/d)	0.016	0.021	0.005	0.017	0.011	0.006
	λ (d)	0.000	0.000	6.610	0.000	0.000	7.020
Modified	R^2^	0.945	0.996	0.993	0.989	0.9931	1.000
Gompertz	AIC	− 41.318	− 53.028	− 86.243	− 52.771	− 48.147	− 152.317
	RMSE	0.059	0.028	0.004	0.029	0.038	0.000
	R_max_ (g/L/d)	0.023	0.037	0.008	0.027	0.018	0.013
	λ (d)	0.000	0.000	6.950	0.000	0.000	7.138
Logistic	R^2^	1.000	1.000	1.000	1.000	1.000	1.000
	AIC	− 19.003	− 12.302	− 89.062	− 35.252	− 33.047	− 89.298
	RMSE	0.238	0.361	0.003	0.086	0.099	0.003
	k (/d)	0.117	0.152	0.026	0.126	0.143	0.017
	n	0.321	0.387	2.471	0.428	0.473	2.519
Fitzhugh	R^2^	1.000	1.000	1.000	0.997	1.000	1.000
	AIC	− 22.384	− 26.659	− 68.128	− 53.608	− 31.549	− 92.298
	RMSE	0.192	0.147	0.011	0.027	0.108	0.002

C3, propionate; C4, butyrate; C6, caproate; k_,_ first-order carboxylate production rate constant; R_max_, maximum rate of carboxylate production; λ, lag phase; R^2^, coefficient of determination; AIC, Akaike Information Criterion; RMSE, root-mean-square-error

The formation of caproate from all other silages, except *Elodea*/wheat straw, was better described by the modified Gompertz model than by the other models. In fact, the carboxylate production could not be predicted by the first-order model. Lag phases of 7.02 and 6.61 days were estimated for caproate formation from sugar beet and cassava leaf silage, respectively. These estimates were consistent with the experimental data, where caproate was only detected from day 7, indicating that the modified Gompertz model adequately predicted the lag phases of these processes. The shape factor from the Fitzhugh model for caproate production from both cassava leaves silage and sugar beet leaves silage was greater than 1.0, which is an indication of the presence of a lag phase. The lag phases of caproate formation obtained in the present study are consistent with a lag phase of 6.905 days reported for caproate formation from swine wastewater using sludge from an anaerobic wastewater treatment plant as inoculum [43]. However, a lag phase of zero was reported for caproate formation from dairy wastewater using brewery wastewater as inoculum [44], indicating that the type of substrate and inoculum used for fermentation influences the lag phase of carboxylate production. The lag phases for propionate and butyrate formation from all silages were zero, as confirmed by the experimental data, which was also observed by Morais et al. [43] and Coelho et al. [44]. In biotechnology processes, a short lag phase is often desired, since it is an indication of a fast start-up of fermentation processes.

The first-order production rate constant (k) is a measure of how fast a given compound is produced (or consumed) during a reaction [17]. For the bottles containing cassava leaf silage, k was higher for butyrate than for propionate, whereas the reverse was the case for sugar beet leaf silage. If the inoculum was adapted for the formation of MCC beyond caproate, odd-numbered MCC would have dominated the systems containing sugar beet leaf silage, while the systems containing cassava leaf silage would have been dominated by even-numbered MCC. Based on the k obtained, the formation of a desired MCC (odd- or even-numbered) can be quantified and maximized by altering certain conditions like reactant concentration, temperature and reactor type. Fogler [17] has reported that the selectivity equation (Eq. 11) can be used to maximize the formation of a desired product:

11 $${S}_{D/U}=\frac{{k}_{D}}{{k}_{U}}{c}^{{\alpha }_{D}-{\alpha }_{U}}$$

where *S* is the instantaneous selectivity, *k* is the first-order production rate constant (/d), *α* is the positive reaction order, and *c* is the substrate concentration (g/L), *D* is the desired product, *U* is the undesired product.

Since the production of both propionate and butyrate was first-order, the selectivity reduces to the ratio of the first-order kinetic constants, indicating that either the temperature, pH, the type of substrates or the reactor type rather than the concentration of fermentation products (lactate and acetate) in the silages probably influenced the selectivity in the present study. Potential approaches to increase the selectivity of even-numbered MCC (as desired product) from the fermentation of sugar beet leaf silage could be operating at a lower pH or using a different type of reactor [17]. Similarly, the maximum carboxylate productivity R_max_ was highest for butyrate in the fermentation of cassava leaf silage, whereas it was highest for propionate in the fermentation of sugar beet leaf silage, which is consistent with the measured concentrations of these two carboxylates. If operating parameters such as inoculum to substrate ratio and pH were optimized, higher values of R_max_ and ρ_max_ for MCCs could be achieved in batch fermentations of the substrates [8, 45]. As also noticed in Fig. 2, there was a sharp drop in butyrate concentration after 21 days during the fermentation of sugar beet leaf silage. Usually, butyrate concentration decreases, because the carboxylate is elongated to caproate. However, in the present study, a corresponding increase in caproate concentration was not observed. It is therefore possible that the sharp drop in butyrate concentration in the system with sugar beet leaf silage was due to the conversion of the carboxylate to products other than caproate.

### Carboxylate formation from sugar beet leaf silage in the semi-continuous systems

**Fig. 3:**
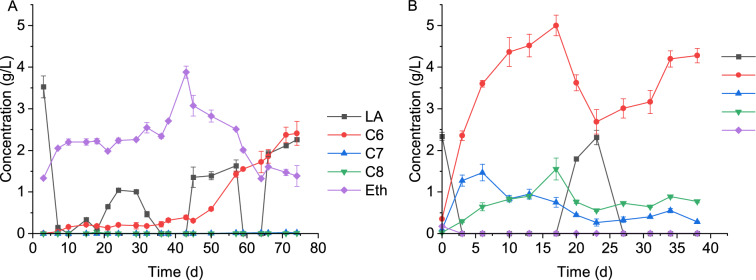
Concentrations of MCCs, lactate and ethanol during the semi-continuous fermentation of sugar beet leaf silage in the experiments **A** E1 and **B** E2. Error bars represent standard deviations. LA, lactate; Eth, ethanol; C6, caproate; C7, enanthate; C8, caprylate

Although a higher yield of caproate was obtained from the fermentation of cassava leaves compared to sugar beet leaf silage, the latter was used as substrate for semi-continuous fermentation due to its availability. Two different experiments, E1 and E2, were conducted to determine the effect of the type of inoculum on MCC production. In the first trial, E1, where only Inoculum 2 was used, very low caproate production of up to 0.15 g/L was observed at the beginning until day 10 (Fig. 3A). The low MCC production in this period resulted from the type of inoculum rather than the low availability of electron donors for CE, as lactate and ethanol were always present in the harvested broth. Limited metabolic capabilities of the inoculum to completely utilize lactate and ethanol were similarly observed in the batch systems. Obviously, Inoculum 1 and Inoculum 2, both being digestates from biogas processes, were not well suited for a fast MCC production start. At several timepoints, the ethanol concentration was above the concentration in the input stream, indicating that this alcohol was produced in the system, which was similarly observed by Chwialkowska et al. [46] in the fermentation of acid whey. The high ethanol concentration may have inhibited caproate producing bacteria [47], resulting in the low initial caproate production.

Despite a substantial butyrate concentration of up to 4.99 g/L (see time-resolved butyrate curves in Figure S2), the caproate concentration remained below 1.0 g/L before day 50 (Fig. 3A). Thereafter, there was a sharp increase up to a maximum concentration of 2.41 g/L on day 74. The increased caproate concentration from day 50 was accompanied by a rapid drop in ethanol concentration, suggesting that ethanol presumably served as electron donor for the CE of butyrate and that microorganisms capable of this CE pathway had grown up.

The concentrations of enanthate and caprylate did not exceed 0.04 g/L throughout the fermentation period, which was similarly observed by Baleeiro et al. [48] in the fermentation of a mixture of lactate and acetate using an inoculum from the same biogas reactor as the one in the present study.

The volume of gas produced in the reactors fluctuated over time, with CO_2_ being the major component, which is consistent with the study of Lambrecht et al. [21] on the fermentation of maize silage. CO_2_ fluctuated between 66.82% and 90.78%, and H_2_ between 6.85% and 28.55% throughout the experiment. Remarkably, only traces of methane (< 1%) were detected in the gas phase of the reactors. The low methane concentration indicates that the methanogens were effectively inactivated by the heat treatment of the inoculum, unlike the batch processes where the methanogens were only partially inactivated or the inactivation was not permanent. The semi-continuous system with its shorter retention time facilitated slow-growing microorganisms to be washed out, whereas in the batch system the longer incubation time made it easier for methanogens to resettle. Traces of oxygen (up to 1.70%) were detected in the reactor headspace, which was probably caused by the daily feeding of the reactors with oxic substrate.

In the second experiment, E2, a mixture of Inoculum 2 and Inoculum 3 was used, the latter being an acclimatized enrichment culture capable of CE. The caproate concentration increased to 2.27 g/L after 3 days and continued to increase steadily over time until it reached a maximum of 5.0 g/L on day 17 (Fig. 3B). A lag phase, as observed in the batch systems and E1, was not present, probably because the microbial community (especially from Inoculum 3) quickly adapted to the conditions. Furthermore, the addition of the adapted Inoculum 3 resulted in ethanol and lactate being completely consumed in the start phase, unlike in E1. Between day 17 and day 31, there was a period of instability characterized by a drop in caproate concentration to 2.68 g/L and an increase in lactate concentration, probably due to a decrease in nitrogen availability resulting from the lack of addition of NH_4_HCO_3_ to the feed. Unlike in E1, enanthate and caprylate were produced in considerable quantities reaching a maximum of 0.75 and 1.55 g/L, respectively, on day 17.

The volume of gas produced also fluctuated over time, with a considerable proportion of methane of up to 30.04% ± 1.78% formed during E2, probably because Inoculum 3 was more resistant to the thermal pretreatment. Hydrogen was mostly not detected, except on day 23 when it rose to 32.71%, at the same time the methane content was about 4% and the caproate concentration dropped. This inverse relationship of hydrogen content and caproate production was also reported by Sträuber et al. [49] for the anaerobic fermentation of maize silage and by Fang et al. [38] for the co-fermentation of mushroom residues and sewage sludge.

### Yield and productivity

The maximum yields of fermentation products and productivities from the semi-continuous processes were calculated and summarized in Table 4. Clearly, the mixture of microbial communities from Inoculum 2 and Inoculum 3 enhanced the yield and productivity of MCCs from ensiled sugar beet leaves. Table 4 shows that the yields and productivities of MCC in E1 and E2 were significantly different (p < 0.05), indicating that CE was more efficient in E2 than in E1. The yield and productivity for MCCs (C6–C8) during E1 were 43.30 g/kgVS and 0.95 g/L/d, respectively, compared to 93.67 g/kgVS and 2.05 g/L/d, respectively, obtained during E2. The selectivity of caproate, enanthate and caprylate during E1 was 12.92, 0.11 and 0.04%, respectively, which was higher than in the batch fermentation, probably due to the adaptation of the microbial community enabled by the extended fermentation time and the semi-continuous operation. During E2, the selectivity of caproate, enanthate and caprylate was 26.86%, 4.03% and 8.33%, respectively, further indicating the positive effect of the enriched microbiome, i.e. Inoculum 3, on the fermentation process. Unlike in the batch systems, the continuous systems produced higher yields of butyrate than propionate, which translated into higher yields of even-numbered MCC than odd-numbered MCC. This can be explained by differences in reactor type and operation as deduced from the selectivity equation (Eq. 8).

**Table 4:** Maximum product yields and productivities for carboxylates in the semi-continuous fermentation of sugar beef leaf silage in experiments E1 and E2

	Yield (g/kgVS)		Productivity (g/L/d)	
	E1	E2	E1	E2
Acetate	104.31	89.57	2.28	1.96
Propionate	11.87	5.72	0.26	0.15
Butyrate	38.62	37.82	0.85	0.83
Valerate	6.27	8.17	0.14	0.18
Caproate	42.74	60.92	0.94	1.33
Enanthate	0.40	10.06	0.01	0.22
Caprylate	0.17	22.69	Negligible	0.50

The substrates used in the present study contained electron donors for microbial CE, so that the addition of these components was not necessary. A comparison of the production of caproate from other substrates without the addition of external electron donors is shown in Table 5. The caproate concentration in the present study was in the same range as for the other substrates, indicating that sugar beet leaf silage is a potentially suitable substrate for MCC production if an adapted inoculum is available.

**Table 5:** Overview of maximum caproate concentrations from anaerobic fermentations of substrates with inherent electron donors for microbial CE

Substrate	Caproate concentration (g/L)	Reactor type	pH	References
Maize silage	3.10	STR	~ 5.0	[49]
Maize silage	6.12	STR	5.5	[21]
Grass silage	4.09	STR	5.5–6.2	[50]
Maize silage	1.40	Batch	~ 4.0	[8]
Sugar beet leaf silage	5.00	STR	5.5	This study
Sugar beet leaf silage	2.41	STR	5.5	This study
Food waste	10.00	Batch	NS	[51]
Acid whey	3.39	UAF	5.0	[52]

STR, stirred tank reactor; UAF, upflow anaerobic filter; NS, not stated

### Microbial community composition and dynamics during semi-continuous fermentation

**Fig. 4:**
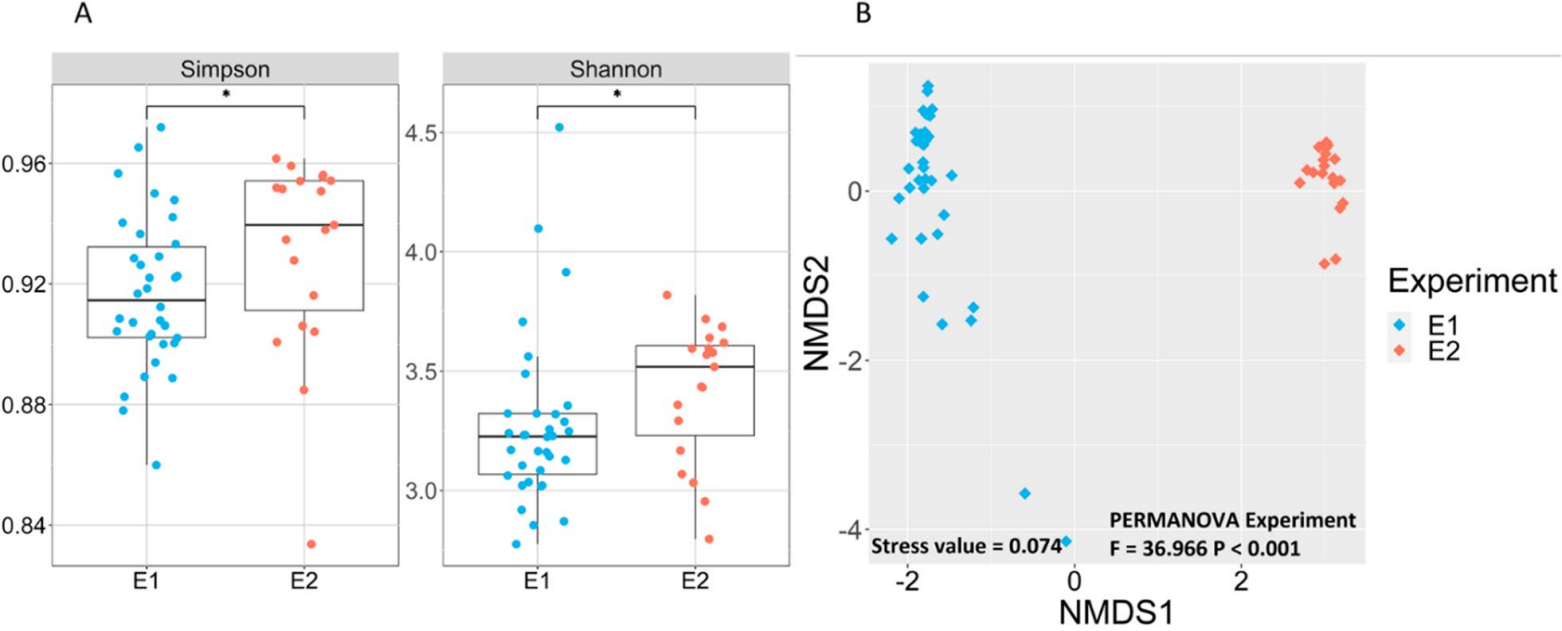
Bacterial alpha- and beta-diversity in the semi-continuous systems E1 and E2. **A** Simpson and Shannon indices. Asterisks indicate significant difference (p < 0.05) based on Wilcox test. **B** Non-metric Multi-dimensional Scaling (NMDS) ordination based on Bray–Curtis distance

The bacterial communities of the semi-continuous systems in experiments E1 and E2 were significantly different as revealed by 16S rRNA gene amplicon sequencing. Communities in E2 presented a higher diversity (*p* < 0.05) compared with E1, considering all timepoints and parallel reactors (Fig. 4A). Beta-diversity was visualized by NMDS (Fig. 4B), showing a clear separation between E1 and E2 samples as confirmed by PERMANOVA (p < 0.001). This result indicates that the use of Inoculum 3 in E2 significantly restructured the reactor microbiome. The parallel community dynamics in duplicate reactors R7 and R8 is also visible in the NMDS plot.

Inoculum 3 originated from the anaerobic fermentation of maize silage, in which the reactor microbiome was demonstrated to produce high titers of MCCs [21]. In contrast, Inoculum 2 was digestate from an anaerobic digester producing biogas. Therefore, the different community composition in E1 and E2 was expected. Since E2 was performed with a merged inoculum of Inoculum 2 and 3, it is clear that Inoculum 3 played a key role in structuring the community. Such bioaugmentation is a strategy used in anaerobic systems to obtain specific functions [53, 54]. Here, the success of bioaugmentation was reflected both in the caproate production and the microbial community composition.

**Fig. 5:**
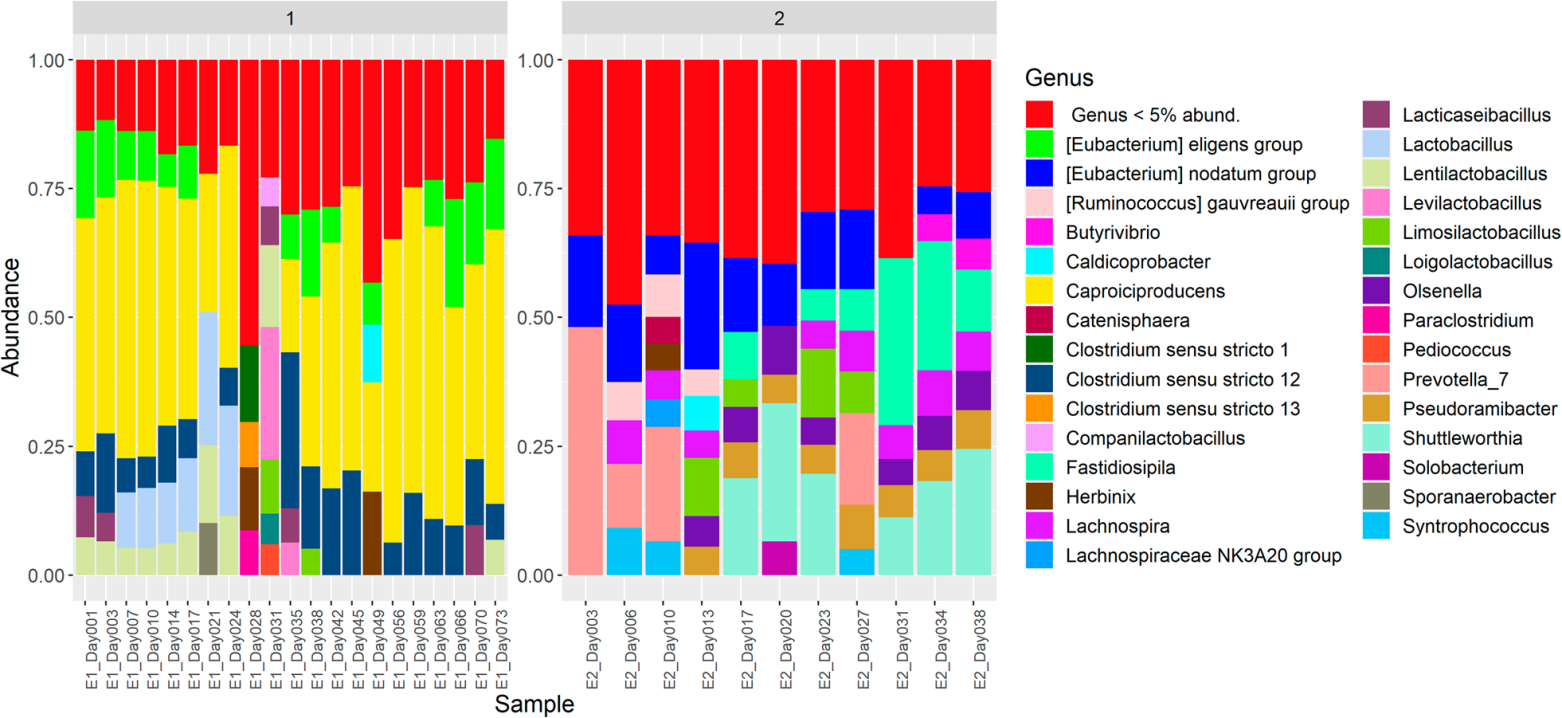
Bacterial community composition of the semi-continuous systems E1 and E2. ASV data of the parallel reactors R7 and R8 were merged as not all sampling days were presented in the individual datasets (shown in Figure S3)

Microbiomes in both experiments harbored mainly bacteria from the phylum Firmicutes, but the taxonomic composition was different on the genus level. In E1, the predominance of *Caproiciproducens* was observed throughout the process (Fig. 5, Figure S3). In the beginning of the E1 process, also lactic acid bacteria of the genera *Lentilactobacillus* and *Lactobacillus* as well as ASVs assigned to *Clostridium* sensu stricto 12 were present in high relative abundance. Interestingly, between days 21 and 38, there was a shift in the community—the relative abundances of *Lentilactobacillus* and *Lactobacillus* first increased (until day 31) and then decreased considerably (below 5% abundance, Fig. 5). On day 31, *Caproiciproducens* was below 5% abundance, but after that, it remained to be dominant.

*Caproiciproducens* is known for its ability to produce caproate [55]. However, in E1, production of caproate was observed only after day 50, and the drop of ethanol concentration around the same time suggests that this was the electron donor for the chain elongation process (Fig. 3). As mentioned before, high ethanol concentrations may inhibit bacteria that can produce caproate. In particular, at ethanol concentrations higher than 4% (*v/v*), caproate production by *Clostridium kluyveri* decreased [47]. In experiment E1, the disturbance between days 21 and 38 may also have played a role in the adaptation of the community to produce caproate.

In E2, the bacterial community was more diverse and mainly composed of the genera *Shuttleworthia*, *Fastidiosipila*, [*Eubacterium*] *nodatum* group, *Lachnospira*, *Olsenella* and *Pseudoramibacter*. They belong to the clostridial families Lachnospiraceae and Eubacteriaceae (Firmicutes), except *Olsenella,* which is a lactic acid bacterium of the class Coriobacteriia (phylum Actinobacteriota). Representants of Lachnospiraceae and Eubacteriaceae were predicted to convert lactate and acetate into MCC by integrative metagenomic, metatranscriptomic, and thermodynamic analyses of lignocellulosic ethanol fermentation conversion residue [56]. These authors specifically reported metagenome recovered genomes (MAGs) that clustered in a phylogenetic analysis with representants of *Shuttleworthia* (Lachnospiraceae) and *Pseudoramibacter* (Eubacteriaceae). Also compared with Lambrecht et al. [21], the study that described the Inoculum 3 previously, many common genera were found.

## Conclusions

Silages with a high content of electron donors such as lactate or ethanol for fueling the microbial CE are good substrates for feasible MCC production processes. In the present study, the modified Gompertz model predicted the formation of caproate from all silages better than the first-order, Logistic and Fitzhugh models. Besides shortening the start-up phase for the production of MCC, higher yields, selectivities and productivities can be achieved when using an enriched microbiome that has been tailored to microbial CE. When digestate from a biogas reactor was used as inoculum, the CE reactors were dominated by *Caproiciproducens,* while a more diverse community with Lachnospiraceae and Eubacteriaceae resulted when an enriched inoculum was used.

### Supplementary Information

Additional file 1.

## Data Availability

The raw sequence reads generated in the present study are deposited in the database of the National Centre for Biotechnology Information (NCBI) under the accession PRJNA926909.
