# Supplementary material for: Medium-chain carboxylates production from plant waste: kinetic study and effect of an enriched microbiome
Source: Biotechnol Biofuels Bioprod. 2024 Jun 12;17:79. doi: 10.1186/s13068-024-02528-y (PMC11167882; doi:10.1186/s13068-024-02528-y)
Supplement: Supplementary file 1 — Additional file 1. [file 13068_2024_2528_MOESM1_ESM.docx]

**Supplementary Information for**

**Medium-chain carboxylates production from plant waste – kinetic study and effect of an enriched microbiome**

Jerome Undiandeye^1,2*^, Daniela Gallegos^1^, Maria L. Bonatelli^3^, Sabine Kleinsteuber^3^, Mohammad Sufian Bin Hudari^4^, Nafi’u Abdulkadir^5,6^, Walter Stinner^1^, and Heike Sträuber^3^

^1^Department of Biochemical Conversion, DBFZ Deutsches Biomasseforschungszentrum gemeinnützige GmbH, 04347 Leipzig, Germany;

^2^Department of Chemical Engineering, University of Port Harcourt, PMB 5323, Port Harcourt, Nigeria;

^3^Department of Microbial Biotechnology, Helmholtz Centre for Environmental Research – UFZ, 04318 Leipzig, Germany;

^4^Department of Isotope Biogeochemistry, Helmholtz Centre for Environmental Research – UFZ, 04318 Leipzig, Germany;

^5^Research Unit for Comparative Microbiome Analysis, Helmholtz Munich, German Research Center for Environmental Health, 85764 Neuherberg, Germany;

^6^Department of Microbiology, Sokoto State University, Sokoto 852101, Sokoto, Nigeria

*****Correspondence: [jerome.undiandeye@uniport.edu.ng](mailto:jerome.undiandeye@uniport.edu.ng)

Figure S1. Change in pH during batch fermentation of MS, maize silage; Elo, *Elodea*/wheat straw silage; Cass, cassava leaf silage; SBL, sugar beet leaf silage; and X+L, xylan+lactate.

Figure S2. Concentration of butyrate (BA), lactate (LA) and ethanol (Eth) during E1 (A) and E2 (B). Error bars indicate standard deviation between replicates.


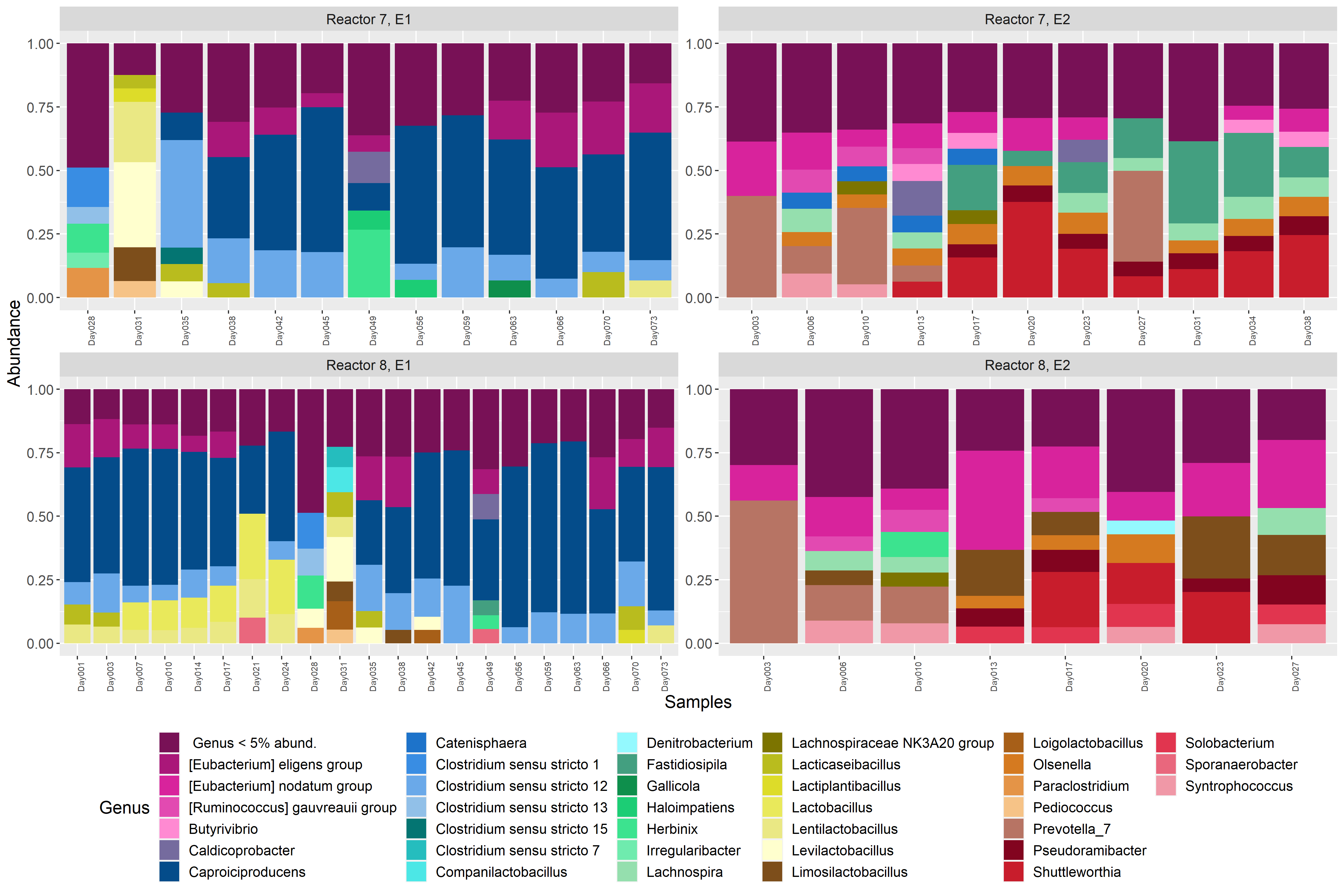


Figure S3. Bacterial community composition of R7 (Reactor 7) and R8 (Reactor 8) during experiments E1 and E2. Reactors 7 and 8 were operated in parallel as biological replicates. ASV data from Reactor 7 in E1 are only available from day 28 on, and ASV data from Reactor 8 in E2 are only available until day 27, which was due to technical problems with DNA extraction, PCR or sequencing for samples from the missing sampling days.
